# Reproducibility in Echocardiographic Assessment of Diastolic Function in a Population Based Study (The STANISLAS Cohort Study)

**DOI:** 10.1371/journal.pone.0122336

**Published:** 2015-04-08

**Authors:** Zied Frikha, Nicolas Girerd, Olivier Huttin, Pierre Yves Courand, Erwan Bozec, Arnaud Olivier, Zohra Lamiral, Faiez Zannad, Patrick Rossignol

**Affiliations:** 1 INSERM, Centre d’Investigations Cliniques 9501, Université de Lorraine, CHU de Nancy, Institut Lorrain du cœur et des vaisseaux, Nancy, France; 2 Service de Cardiologie, Institut Lorrain du Cœur et des Vaisseaux, Centre Hospitalier Universitaire de Nancy, Nancy, France; 3 Fédération de cardiologie, European Society of Hypertension Excellence Centre, hôpital de la Croix-Rousse, hospices civils de Lyon, 103, Grande Rue de la Croix-Rousse, 69004 Lyon, France; 4 Génomique fonctionnelle de l'hypertension artérielle, EA 4173, université Claude-Bernard Lyon 1, 69100 Villeurbanne, France; 5 Hôpital Nord-Ouest, 69400 Villefranche-sur-Saône, France; Providence VA Medical Center and Brown University, UNITED STATES

## Abstract

**Introduction:**

There is limited evidence regarding intra-observer and inter-observer variations in echocardiographic measurements of diastolic function. This study aimed to assess this reproducibly within a population-based cohort study.

**Methods:**

Sixty subjects in sinus rhythm were randomly selected among 4^th^ visit participants of the STANISLAS Cohort (Lorraine region, France). This 4^th^ examination systematically included M-mode, 2-dimensional, DTI and pulsed-wave Doppler echocardiograms. Reproducibility of variables was studied by intra-class correlation coefficients (ICC) and Bland Altman plots.

**Results:**

Our population was on average middle-aged (50 ± 14y), overweight (BMI = 26 ± 6kg/m^2^) and non-smoking (87%) with a quarter of the participants having self-declared hypertension or treated with anti-hypertensive medication(s). Intra-observer ICC were > 0.90 for all analyzed parameters except for left ventricular ejection fraction (LVEF) which was 0.89 (0.81–0.93). The mean relative intra-observer differences were small and limits of agreement of relative differences were narrow for all considered parameters (<5% and <15% respectively). Inter-observer ICC were > 0.90 for all analyzed parameters except for LVEF (ICC = 0.87) and both mitral and pulmonary A wave duration (0.83 and 0.73 respectively). The mean relative inter-observer differences were <5% for all parameters except for pulmonary A wave duration (mean difference = 6.5%). Limits of agreement of relative differences were narrow (<15%), except for mitral A wave duration and velocity (both <20%) as well as left ventricular mass and pulmonary A wave duration (both <30%). Intra-observer agreements with regard to the presence and severity of diastolic dysfunction were excellent (Kappa = 0.93 (0.83–1.00) and 0.88 (0.75–0.99), respectively).

**Conclusion:**

In this validation study within the STANISLAS cohort, diastolic function echocardiographic parameters were found to be highly reproducible. Diastolic dysfunction consequently appears as a highly effective clinical and research tool.

## Introduction

Heart failure (HF) with preserved left ventricular (LV) ejection fraction (EF) is a progressive disorder characterized by impaired LV relaxation, increased LV stiffness, increased interstitial deposition of collagen and modified extracellular matrix proteins. HF with preserved LVEF (HFPEF) is frequently referred to as diastolic heart failure because of the presence of diastolic LV dysfunction resulting from slow LV relaxation and increased LV stiffness [1]. Because the process of LV myocardial remodeling invariably begins before the onset of symptoms, current guidelines place special emphasis on the detection of subclinical diastolic dysfunction and the timely identification of patients who are at risk of developing overt HF [2].

The gold standard for assessing diastolic function remains pressure-volume relationship. However, this invasive approach is never used in clinical practice. An extensive non-invasive assessment of diastolic function is now possible, and its clinical evaluation is mostly based on echocardiographic parameters [3]. Indeed, echocardiography provides valuable information regarding LV diastolic function in addition to classical M-mode and 2D echocardiography and pulsed-wave Doppler. Nevertheless, these techniques remain complex since no single measurement reflects diastolic function. Thus, a comprehensive assessment of a number of variables is required to evaluate diastolic function as accurately as possible [3–5].

The value of a given parameter is based on its ability and reproducibility to correctly predict a clinical entity. Therefore, each parameter of diastolic function should be obtained in a standardized and reliable manner.

Verifying the reliability of each parameter of diastolic function is a crucial aspect in clinical practice because it assists clinicians in establishing the diagnosis of HFPEF and decision-making especially in situations of discordant echocardiographic parameters. In epidemiology, accuracy of a measurement process is defined as the degree to which a measurement represents the true value of the parameter being measured. Reproducibility refers to the capacity to produce the same result on each occasion under identical conditions [6]. A measurement process is accurate, or unbiased, if the expected value of the measurement is the true value of the parameter being estimated. Presently, available results from other studies on intra-observer and inter-observer variations of all echocardiographic measurements for diastolic function are limited.

Recently, an array of HF biomarkers has emerged, each reflecting different pathophysiological processes in the development and progression of HF, namely myocardial insult, inflammation and remodeling. The assessment of a new biomarker should be made on a wide range of patients and in typical conditions of its intended use. The diagnostic methods used to detect diastolic HF should thus be contemporary, rigorous, standardized and fair. Consequently, we endeavored to assess LV diastolic function using an accurate measurement process.

The present study was aimed to validate a standardized process of echocardiographic assessment of diastolic function in a population-based study by analyzing both intra-observer and inter-observer reproducibility of all parameters of diastolic function.

## Methods

### Study population

The STANISLAS Cohort is a single-center familial longitudinal cohort comprised of 1006 families (4295 subjects) from the Nancy region recruited in 1993–1995 at the Centre for Preventive Medicine. This cohort was established with the primary objective of investigating gene-gene and gene-environment interactions in the field of cardiovascular diseases. The families were deemed healthy, free of declared acute and/or chronic illness, in order to assess the effect of genetics on the variability of intermediate phenotypes on the transition toward pathology.

From 2011 to 2014, 1200 survivors of the original cohort underwent their 4^th^ examination at our department. M-mode, 2-dimensional, DTI and pulsed-wave Doppler echocardiograms were routinely performed at the 4^th^ examination. For the present study, 60 subjects in sinus rhythm were randomly selected from the STANISLAS Cohort study.

The research protocol was approved by local Ethics Committee (Comité de Protection des Personnes Est III—Nancy—France) and all study participants gave a written informed consent to participate. The informed written consent was previously approved by the local Ethics Committee.

### Study design

#### Echocardiographic image acquisition

Examinations were performed by an experienced echocardiographer in the left lateral decubitus position with a commercially available standard ultrasound scanner (Vivid 9, General Electric Medical Systems, Horten, Norway) using a 2.5 MHz phased-array transducer (M5S). The echo/Doppler examination included parasternal long- and short axis views and three standard apical views. For each view, at least three consecutive cardiac cycles were recorded during quiet respiration.

From the three apical views, separate M-mode and color tissue Doppler acquisitions were recorded. The Doppler pulse repetition frequency was 1 kHz. Pulsed-wave (PW) Doppler was performed in the apical 4-chamber view to obtain mitral inflow velocities to assess LV filling. Recording for isovolumic relaxation time measurement was obtained by simultaneous recording of the aortic and mitral flows. PW Doppler Tissue Imaging (DTI) was performed in the apical views to acquire mitral annular velocities. The sample volume was positioned at 1 cm of the septal and lateral insertion sites of the mitral leaflets and adjusted as necessary (usually 5–10 mm) to cover the longitudinal excursion of the mitral annulus in both systole and diastole. Pulmonary venous flow velocities were recorded into the right upper pulmonary vein from the apical 4-chamber view. Color flow imaging was used for the proper location of the sample volume and the 2-mm sample volume was placed 5 mm into the pulmonary vein for optimal recording of the spectral waveforms.

All acquired images and media were stored on a secured network server as digital clips, using a unique identification number and analyzed on a dedicated workstation (EchoPAC PC, version 110.1.0, GE Healthcare).

#### Reproducibility analysis

Two experienced physician echocardiographers (Z.F. and O.H.), blinded to each other and to the demographic parameters, performed a separate complete analysis of diastolic function of the 60 participants. The first physician echocardiographer (Z.F.) re-analyzed the entire data set of all 60 study patients a second time. The re-analyses were performed in random order after a period of 4 weeks. The first reading of the first analyzer (Z.F.) was considered for the statistical analysis of inter-observer variability.

Intra-analyzer reproducibility was defined as the reproducibility calculated by one of the echocardiographer's re-analysis of his own recordings (Z.F.). Inter-analyzer reproducibility was defined as the reproducibility calculated by the two echocardiographers’ analyses of the same set of recordings.

#### Examination of diastolic function

LV mass: LV mass was measured from 2-dimensional (2D) echocardiography, using the recently published guidelines of the American Society of Echocardiography. We measured the septal wall thickness (SWT), posterior wall thickness (PWT) and LV internal diastolic diameter (LVID) from the parasternal 2D long axis view. These measurements were subsequently used in the cube-function formula for ASE guidelines to calculate LV mass [7].

LA Volume: The measurement of left atrial (LA) volume was obtained from the biplane method of disks (modified Simpson’s rule) using apical 4-chamber (A4C) and apical 2-chamber (A2C) views at ventricular end systole [7].

Mitral Inflow: The following parameters were studied: peak early filling (E-wave) and late diastolic filling (A-wave) velocities, E/A ratio, deceleration time (DT) of early filling velocity and mitral A-wave duration obtained at the tip of the mitral valve leaflets.

Pulmonary Venous Flow: Measurements included peak systolic (S) velocity, peak anterograde diastolic (D) velocity, the peak reversal A-wave (Ar) velocity in late diastole and the duration of Ar velocity,

Tissue Doppler Annular Early and Late Diastolic Velocities: Measurements included systolic (S), early diastolic (Ea) and late diastolic velocity (Aa) of the septal and lateral mitral annulus [8].

Assessment of left ventricular diastolic dysfunction was made according to the recommendations of the American Society of Echocardiography and the Committee of the European Association of Echocardiography [3] using the following grading scheme: mild or grade I (impaired relaxation pattern), moderate or grade II (PNF) and severe (restrictive filling) or grade III [3]. Grades II and III were grouped together because of the relatively small number of subjects in these 2 groups.

#### Assessment of LVEF

LVEF was calculated using the biplane Simpson’s method.

### Biostatistics methods

The reproducibility of continuous variables is expressed by the intra-class correlation coefficients (ICC). As all analyzed parameters constituted continuous variables, the inter-observer and intra-observer agreements were estimated using the ICC. Agreement was classified as poor (ICC = < 0.40), fair to good (ICC = 0.40–0.75) or excellent (ICC > 0.75).

For descriptive purposes, the Bland Altman plots report the means of the ratings for each patient by two raters vs. the differences in ratings. Mean absolute error was calculated as the mean of the absolute difference between the two sets of observations [9]. The mean relative error was calculated as the absolute difference between the two sets of observations divided by the mean of the observations. To assess the degree of agreement between techniques, 95% confidence limits (i.e. mean absolute or relative error±1.96*SD) were calculated and plotted on each graph.

P values < 0.05 were considered statistically significant. All analyses were performed using SAS version 9.3 (SAS Institute Inc., Cary, N.C., USA) and SPSS 21 (SPSS, Chicago, IL, USA).

## Results

Overall, our study population was middle-aged, overweight and non-smoking (13%), with a quarter of the participants having self-declared hypertension or being on anti-hypertensive medication (Table 1).

**Table 1:** Patient characteristics.

**Characteristics**	
Female (%)	33 (55%)
Age (years, mean±SD)	50 ± 14
Height (m, mean±SD)	1.68 ± 0.09
Weight (Kg, mean±SD)	73.55 ± 19.19
BMI (Kg/m^2^, mean±SD)	26 ± 6
Current tobacco use (%)	8 (13%)
Hypertension (%)	15 (25%)

Legend: BMI, body mass index.

### Intra-observer reliability

The intra-observer ICC coefficients were >0.90 for all analyzed parameters except for LVEF which was 0.89 (0.81–0.93) (Table 2).

**Table 2:** Intra- and inter-observer intra-class correlation coefficients for echocardiographic variables.

	Intra-observer ICC		Inter-observer ICC	
LVEF (%)	0.89	0.81–0.93	0.87	0.78–0.93
LV mass (g)	0.98	0.97–0.99	0.95	0.91–0.97
LA volume (ml)	0.99	0.99–1.00	0.99	0.98–1.00
E wave (m/s)	0.98	0.97–0.99	0.97	0.96–0.99
A wave (m/s)	0.98	0.97–0.99	0.97	0.94–0.98
Septal Ea Velocity (cm/s)	0.99	0.99–1.00	0.98	0.97–0.99
Lateral Ea Velocity (cm/s)	0.99	0.99–1.00	0.99	0.98–1.00
Deceleration time (ms)	0.98	0.97–0.99	0.96	0.94–0.98
A wave duration (ms)	0.94	0.91–0.97	0.83	0.72–0.90
Pulmonary reversal A wave duration(ms)	0.96	0.93–0.98	0.73	0.55–0.84

Legend: ICC, Intra-class correlation coefficients; LVEF, left ventricular ejection fraction; LV, left ventricular; LA, left atrial.

**Fig 1:**
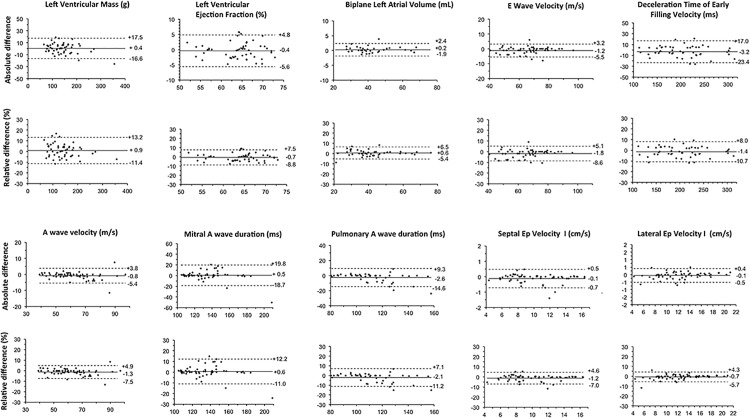
Bland-Altman plots of intra-observer agreement for left ventricular ejection fraction, LV mass, LA Volume, E-wave, E-wave deceleration time, A-wave velocities, A-wave and pulmonary vein reversal A duration, as well as *s*eptal and lateral Ep Velocity.

The mean relative intra-observer differences were <5% for all parameters (Fig 1).The limits of agreement of relative differences for all analyzed parameters were small (2 SDs <15%), indicating a high reproducibility of within-observer measurements. Importantly, the reproducibility of both mitral and Pulmonary A wave duration was excellent (ICC = 0.94 and 0.96 respectively).

### Inter-observer reliability

The inter-observer ICC coefficients were >0.90 for most analyzed parameters (Table 2). Slightly lower ICC were observed for LVEF (ICC = 0.87) and both mitral and pulmonary A wave duration (0.83 and 0.73 respectively). Of note, the ICC observed for pulmonary A wave duration was lower than 0.80.

**Fig 2:**
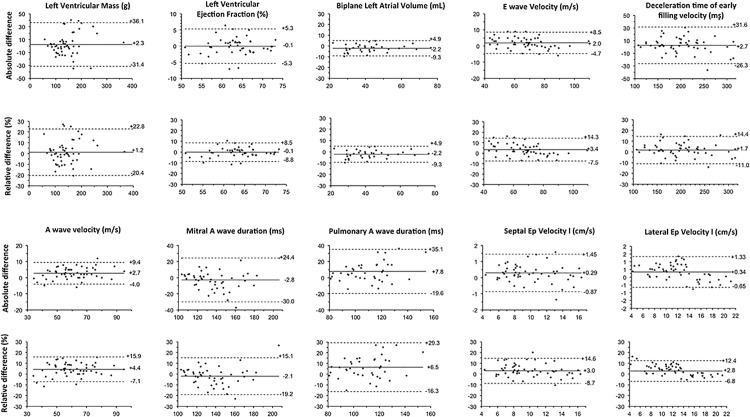
Bland-Altman plots of inter-observer agreement for left ventricular ejection fraction, LV mass, LA Volume, E-wave, E-wave deceleration time, A-wave velocities, A-wave and pulmonary vein reversal A duration, as well as septal and lateral Ep Velocity.

As expected, most limits of agreement were larger for inter-observer comparisons than that observed for intra-observer comparisons (Fig 2).The mean relative inter-observer differences were <5% for all parameters except for the velocity time integral of pulmonary venous flow (mean relative difference = 6.5%). Limits of agreement of relative differences were small (2 SDs <15%), except for mitral A wave duration and velocity (both <20%) as well as for left ventricular mass and pulmonary A wave duration (both <30%).

### Intra-observer and inter-observer agreement

Intra-observer agreement with regard to both the presence of diastolic dysfunction and the severity of diastolic dysfunction was excellent (respectively Kappa = 0.93 (0.83–1.00) and 0.88 (0.75–0.99) (Table 3). Inter-observer agreement with regard to the presence of diastolic dysfunction was excellent whereas it was good with regard to the severity of diastolic dysfunction.

**Table 3:** Kappa measurements of intra- and inter-observer agreement with regard to diastolic dysfunction.

** **	**Kappa (95% CI)**	
	**Diastolic dysfunction**	**Diastolic dysfunction grades (0, 1, ≥2)**
**Intra-observer**	0.93 (0.83–1.00)	0.88 (0.75–0.99)
**Inter-observer**	0.88 (0.76–1.00)	0.74 (0.59–0.89)

Legend: 95% CI: 95% confidence interval

## Discussion

In the present study, we analyzed the reproducibility of all previously validated Doppler indices of diastolic function. The main finding of this study was that both inter- and intra-observer reproducibility was excellent for most diastolic function parameters with limits of agreement sufficiently narrow to conclude that the fluctuation in measurements of diastolic function is unlikely to have a noteworthy impact on diastolic function classification. We also report excellent inter- and intra-observer agreements with regard to diastolic dysfunction identification and grading. Overall, these results thus qualify the STANISLAS cohort for further research in the field of diastolic function, including the validation of biomarkers to predict and classify echocardiographic diastolic function.

While prior investigations have assessed the magnitude of variability parameters of diastolic function, these measurements were performed separately and in limited numbers of normal subjects in clinical setting [7,10–13].The present study is the first to evaluate the reproducibility of all parameters of diastolic function in an unselected population-based cohort leading to a global and better assessment of diastolic function. In addition, the current study is unique in analyzing the variability of Doppler diastolic function indices in a large random sample of subjects in a population-based cohort of initially healthy subjects.

### Intra-observer reliability and agreement

The intra-observer reproducibility for LV systolic function observed in the present study (ICC = 0.89) was globally better than that previously reported. In the study of Palmieri et al., a poor reliability was observed for measurements of LV systolic function (ICC = 0.65)[14]. In contrast, Otterstad et al. demonstrated that differences between repeated echo recordings and repeated video measurements were small and not statistically significant [15]. Furthermore, the findings herein are in agreement with previous studies reporting a high intra-observer reproducibility of diastolic filling and LV mass measurements [7]. In the study of Galderisi et al.[16], intra-observer correlation coefficients were close to 0.90 while the intra-observer percent bias was <10% for Peak velocity of the E and A wave. In contrast with our study, the intra-observer correlation coefficient reported by these authors was <0.90 for deceleration time while the intra-observer relative bias was >10%. Meanwhile, Nagueh et al. [8] reported a mean percentage of intra-observer error of 5% ±4 for E / e'. In our study, the mean relative intra-observer differences were systematically <5% for these parameters. With regard to LV mass, Grandits et al. [13] reported means and SDs of intra-observer difference in LVM of -0.0 and 20.4 g respectively whereas Palmieri et al.[14] showed an intra-observer difference in LV mass of -1.7 ±19.8 g for absolute values with a high reliability for LV mass as estimated by an ICC = 0.93. In our study, we detected small means and SDs of absolute intra-observer differences for LV mass (0.4g and 17.5, respectively). Consequently to these results, our measurement protocol enabled a consistent reading of stored echocardiographic data. This very good intra-observer reliability in turn resulted in an excellent agreement with regard to identification and grading of diastolic dysfunction.

### Inter-observer reliability and agreement

The present study also yielded superior results than previous studies regarding inter-observer reproducibility of systolic and diastolic function as well as LV mass measurements. Indeed, in the study of Otterstad et al. [15], there was a systematic difference between the two investigators in their measurements of left ventricular ejection fraction. As for diastolic function, Galderisi et al. [16] reported an inter-observer percent bias >10% for time velocity integral E and deceleration time, in addition to a relatively weak inter-observer agreement (ICC = 0.76) for deceleration time. Nagueh et al. [8] reported a mean percentage of inter-observer error of 6% ± 5 for E / e'. In contrast, Kuznetsova et al. [17], upon assessing inter-observer measurements of 17 tracings performed by one experienced recorder, reported an inter-observer mean absolute difference of 0.05 cm/s, 0.02 cm/s, 0.31 ms and 1.42 ms for E wave velocity, A wave velocity, mitral and pulmonary A wave duration, respectively. In our study, the mean relative inter-observer differences were <5% for these parameters, which are in keeping with the results reported by Kuznetsova et al. [17].

LV Linear Dimensions and Wall Thickness are used for virtually all clinical trials that incorporate echocardiography. Linear dimensions are obtainable from correctly-aligned 2D and M-mode images. M-mode recordings provide better temporal resolution for accurate timing of motion of cardiac walls and valves, whereas 2D provides better spatial orientation. With respect to LV mass, Valdez et al. [18] found statistically significant inter-observer differences in measurements of end-diastolic septal thickness, end-diastolic and end-systolic LV posterior wall thicknesses as well as end-diastolic and end-systolic LV diameters by 3 observers on 20 echocardiograms. However, the maximum mean difference was 2 mm which would translate into a moderate error in terms of LV mass. Finally, Grandits et al. [13] reported a mean ± SD of 7.9 ± 34.7 g for inter-observer difference between the 2 cardiologists. In our study, we detected a small mean of inter-observer difference with moderately wide limits of agreement for LV mass (<5% and <30% respectively).This overall very good inter-observer reliability resulted in a good to excellent agreement with regard to diastolic dysfunction identification and grading (Kappa = 0.88 (0.76–1.00) and 0.74 (0.59–0.89), respectively). Along with the aforementioned excellent intra-observer agreement, this level of inter-observer agreement qualifies diastolic dysfunction as a relevant and worthy inclusion criterion and outcome in clinical research.

In our study, overall inter-observer agreement in the assessment of diastolic dysfunction classification was better than the results of a multicenter study reported by Unzek et al.[18–21] [18–21]. In this previous study [18–21], a complete diastolic assessment was obtained from the echocardiographic data of 20 patients and interpreted by 14 experts in 8 countries using the current ASE/EAE recommendations for diastolic assessment [3]. The agreement between raters for assessing diastolic class was moderately lower (Fleiss kappa = 0.62) than that observed in the present study (Kappa = 0.74). In our opinion, the lower agreement reported by Unzek et al. is probably the consequence of the multicenter setting of this previous study, such that the use of the same current ASE/EAE algorithm, even by expert echocardiographers, may still have contained sufficient ambiguity to lead to inconsistent final results. In contrast, in our single-center study, with readers working in the same echocardiography laboratory and applying the same echocardiography working standards, the use of the ASE/EAE algorithm resulted in very similar decision-making processes regarding diastolic dysfunction classification. Yet, if high reproducibility can be achieved in a single center, a harmonization process could be undertaken across centers to achieve a better multi-center agreement with regard to diastolic function grading. Such a process could prove valuable in harmonizing the diagnosis of diastolic dysfunction and heart failure with preserved ejection fraction. However, the modalities of such a harmonization process remains to be determined in further studies.

### Large difference in pulmonary A wave duration assessment

The present study revealed a low inter-observer correlation for pulmonary A wave duration with a relatively weak inter-observer agreement (ICC = 0.73) and mean relative inter-observer differences >5%. This result can be explained by the difficulty in the acquisition of this parameter. Indeed, one of the important limitations in interpreting pulmonary venous flow is the difficulty in obtaining high-quality recordings suitable for measurements. This is especially true for pulmonary A wave duration because of low-velocity wall motion artifacts created by atrial contraction that obscure the pulmonary flow velocity signal. Importantly, this variable involved in the determination of filling pressures is not present in the ASE/ESC algorithm of diastolic dysfunction [3]. Consequently, the poor reproducibility of A wave duration would thus impact on the qualification of patients for HF with preserved LVEF rather than on the quantification of diastolic dysfunction according to the algorithm of the ASE/ESC [3].

### Differences in imaging validation protocols

Most previously-conducted studies varied considerably in terms of study design and analysis methods, making direct comparison difficult [18–21]. There are many ways to identify as well as control acquisition and measurement variability; however, successful quantification of diastolic function is entirely dependent on image quality. Each echocardiography core lab must be able to demonstrate the efficiency of its work, including operational processes and accuracy of echocardiographic measurement and interpretation. In our study, the physicians and technicians ensured high-quality acquisition data such that accurate quantitative results could be obtained.

Our analysis results compared favorably with other studies. These good results are explained by the design of our protocol based on reanalysis of the same recorded images collected previously by trained operators with a high-resolution system. According to Otterstad et al. [15], the major component of the random variation in left ventricular volumes and function was repeated echo recordings whereas repeated video measurements and inter-observer variability had a lesser impact. In the present study, we also assessed the magnitude of variability of Doppler diastolic measurements in a relatively large number of healthy patients, which likely increased the reproducibility of our measurements.

### Clinical perspectives

The successful application of echocardiography in clinical heart failure trials depends on careful attention to quality control, including echocardiographic acquisition and interpretation harmonization by an experienced and competent core laboratory. The present study thus focused on specific echocardiographic parameters to obtain positive evidence of abnormal LV relaxation, filling, diastolic distensibility and diastolic stiffness, in addition to assessing the reliability of specific parameters needed to classify the patient according to the diagnostic flowchart of the Echocardiography Associations of the European Society of Cardiology.

Natriuretic peptides and echocardiography are the main tools to detect HF. In the setting of HF with preserved LVEF, echocardiography is of key importance, especially in older patients in whom the use of natriuretic peptides has some limitations. However, access to echocardiography is sometimes difficult and/or long and further compounded by the fact that echocardiographic measurements can show high individual variability in addition to varying day to day in the same patient with changes in preload, afterload and heart rate. We are currently conducting, within the HOMAGE FP7 program (http://www.homage-hf.eu/), a biomarker validation (algorithm, program) for the prediction of diastolic dysfunction and presymptomatic heart failure. Obviously, such a validation can solely be obtained for a reproducible echo assessment of diastolic function.

In the current validation study, all of the echocardiographic parameters assessing diastolic function were found to be highly reproducible in the STANISLAS cohort. Given this high reproducibility of diastolic function assessment, and more specifically in this population-based study, we are thus confident of being able to validate a number of new biomarkers to predict diastolic dysfunction.

In addition, the high reproducibility of diastolic function parameters combined with the observed good agreement relative to diastolic dysfunction identification and grading has important practical clinical implications. We provide strong evidence to support that echocardiographic assessment of diastolic function is a reliable and trustworthy clinical tool in terms of metronomic properties.

### Limitations

The results of our study are based on patients with normal sinus rhythm, which may limit the generalizability of our findings to subjects in atrial fibrillation. Further studies are warranted to assess the reproducibility of diastolic dysfunction grading in patients with atrial fibrillation.

### Conclusion

In the present study, we have validated an operating process for echocardiographic image analysis and quality control measurement for the assessment of diastolic function in patients with sinus rhythm of the STANISLAS cohort study. The excellent results observed in our analysis compared with other studies are explained by the design of our protocol and the single-center nature of our study. This excellent reproducibility will enable the validation of new biomarkers within EU- and French-funded research programs. In addition, in light of these results, diastolic dysfunction echocardiographic assessment thus appears as a highly reliable clinical and research tool.
